# Treatment of sleep-disordered breathing in opioid users with adaptive servo-ventilation: a subgroup analysis of the European READ-ASV registry

**DOI:** 10.5664/jcsm.11652

**Published:** 2025-07-01

**Authors:** Jean-Louis Pepin, Adam V. Benjafield, Oliver Munt, Holger Woehrle, Raphael Heinzer, Michael Arzt, for the READ-ASV Investigators

**Affiliations:** ^1^ University Grenoble Alpes, INSERM U1300, CHU Grenoble Alpes, HP2 Laboratory, Grenoble, France; ^2^ ResMed Science Center, Sydney, New South Wales, Australia; ^3^ ResMed Science Center, Munich, Germany; ^4^ Sleep and Ventilation Center Blaubeuren, Lung Center Ulm, Ulm, Germany; ^5^ Centre d`Investigation et de Recherche sur le Sommeil, Centre Hospitalier Universitaire Vaudois (CHUV), Lausanne, Switzerland; ^6^ Department of Internal Medicine II, University Hospital Regensburg, Regensburg, Germany

**Keywords:** central sleep apnea, opioids, adaptive servo-ventilation, quality of life, symptomatic

## Abstract

**Study Objectives:**

Central sleep-disordered breathing (SDB) is associated with negative health outcomes. Intake of opioids influences stability of breathing and can cause central apneas. Screening for and treatment of SDB is recommended for individuals who use opioids. This subanalysis of the READ-ASV (Registry on the Treatment of Central and Complex Sleep-Disordered Breathing with Adaptive Servo-Ventilation) investigated the effects of adaptive servo-ventilation therapy (ASV) on SDB symptoms in people using opioids.

**Methods:**

Patients initiated on ASV who reported intake of opioids at baseline were included in this analysis of real-world registry data. Patients were prospectively followed-up for 12 months. Disease-specific quality of life was assessed with the Functional Outcomes of Sleep Questionnaire. Sleepiness was measured with the Epworth Sleepiness Scale. Symptomatic patients were defined as having a Functional Outcomes of Sleep Questionnaire score of < 17.9 and an Epworth Sleepiness Scale score of > 10.

**Results:**

Eighty-six patients who reported opioid use were included. The population had severe SDB (median apnea-hypopnea index: 55 events/h), the majority (n = 75 [87%]) had comorbidities, and 81.6% (40/49 with follow-up available questionnaires) were symptomatic at baseline. ASV effectively treated SDB (residual median apnea-hypopnea index from device data [apnea-hypopnea index flow]: 1.5 events/h). The Functional Outcomes of Sleep Questionnaire (+1.4 points; *P* = .003) and Epworth Sleepiness Scale (−3 points; *P* = .029) scores improved significantly at follow-up compared with baseline, and improvements in disease-specific quality of life were more pronounced in symptomatic patients.

**Conclusions:**

ASV treatment of central breathing disorders in individuals using opioids resolved SDB and was associated with significant improvements in disease-specific quality of life and sleepiness. ASV treatment may therefore be an actionable intervention to counteract the negative effects of opioids on SDB and quality of life.

**Clinical Trial Registration:** Registry: ClinicalTrials.gov; Name: Registry on the Treatment of Central and Complex Sleep-Disordered Breathing With Adaptive Servo-Ventilation (READ-ASV); URL: https://www.clinicaltrials.gov/study/NCT03032029; Identifier: NCT03032029.

**Citation:**

Pepin J-L, Benjafield AV, Munt O, Woehrle H, Heinzer R, Arzt M; for the READ-ASV Investigators. Treatment of sleep-disordered breathing in opioid users with adaptive servo-ventilation: a subgroup analysis of the European READ-ASV registry. *J Clin Sleep Med*. 2025;21(7):1227–1232.

**BRIEF SUMMARY**

**Current Knowledge/Study Rationale:** Opioid use is widespread and opioid usage is known to induce or worsen central sleep-related breathing disturbances. The impact of adaptive servo-ventilation positive airway pressure therapy on health-related quality of life in individuals taking opioids is unknown.

**Study Impact:** This real-world data showed that, in addition to controlling respiratory events during sleep, treatment with adaptive servo-ventilation had a clinically relevant impact on disease-specific quality of life and daytime sleepiness in people who take opioids. Adaptive servo-ventilation is therefore an important treatment option in this setting.

## INTRODUCTION

Opioids are used in therapy of acute and chronic pain.^1^ Although opioids are effective at controlling pain, opioid use disorder has been increasing in prevalence and is responsible for a huge health care and economic burden, and high rates of mortality.^2^^–^^4^ In addition, opioids can induce or worsen sleep-related breathing disturbances,^5^^,^^6^ primarily, central sleep apnea (CSA).

In CSA, airflow is reduced or ceases due to a lack of respiratory drive or instability in respiratory control.^6^ This type of sleep-disordered breathing (SDB) often occurs in patients with heart failure.^7^^,^^8^ The second most common cause of CSA is the use of opioids.^9^ Estimates of the proportion of people receiving pain therapy with opioids who have CSA range from 20 to 60%.^10^^–^^13^ Although the opioid crisis in Europe is not as extensive as that in the United States, a considerable number of individuals with pain are treated with opioids and could thus experience treatment-related side effects.^14^ In addition, the effects of opioid medication often overlap with the effects of disturbed breathing and CSA-related symptoms, such as sleepiness.^15^

Awareness of SDB in opioid recipients and screening for CSA is recommended.^16^ This allows the medication dosage to be adjusted to achieve effective pain control while minimizing the adverse effects of therapy, including the impact on breathing, and the selection of an appropriate therapy for residual SDB.^17^ One effective therapy option for CSA is adaptive servo-ventilation (ASV), a bilevel positive airway pressure therapy that adapts applied pressures to stabilize the breathing pattern.^18^^–^^21^ In addition to effectively treating CSA, improvements in quality of life outcomes during ASV therapy have been reported in a large European cohort study.^22^ Although ASV has been used to treat opioid-associated CSA,^23^^,^^24^ the effects of ASV on symptoms and health care–related quality of life in this patient population are not known.

This subanalysis of the Registry on the Treatment of Central and Complex Sleep-Disordered Breathing with Adaptive Servo-Ventilation (READ-ASV) evaluated the effects of ASV on disease-specific quality of life and sleepiness in a cohort of patients with CSA who reported use of opioids.

## METHODS

### Study design

The READ-ASV registry is a European observational study that included 801 patients enrolled in sleep facilities in Germany, Switzerland, France, Denmark, Portugal, and Spain.^22^^,^^25^ Briefly, data from adult individuals with a new prescription of ASV therapy were collected between September 2017 and March 2021. Ethical approval from an ethics committee at each investigator`s site was obtained before study start. Patients were informed about the procedures of the study and consented to the use of their data for research purposes. The current analysis includes READ-ASV registry participants for whom opioid usage was recorded at baseline.

### Participants

Full details of the protocol and overall study population have been previously described.^25^ Briefly, ASV-naive individuals with an indication for treatment with ASV according to applicable medical guidelines^6^ and prescription of an eligible ASV device who started therapy ≤ 7 days before inclusion were enrolled. Patients with contraindications to ASV therapy were excluded. SDB was diagnosed using polysomnography or cardiorespiratory polygraphy according to routine clinical practice at the enrolling center. Only respiratory data from the diagnostic phase were collected. No data on sleep parameters were included in the study’s electronic Case Report Form. At the initial visit, the investigator documented the concomitant treatments being used by participants, including opioids (this was recorded in the electronic Case Report Form as yes/no for each major drug class). This subanalysis included patients who reported the use of opioid medication at baseline. Since opioid medication was not an inclusion criterion in the main registry, no details on the specific opioids being used or opioid dosages were collected.

### Outcomes

End points were changes in scores of the Functional Outcomes of Sleep Questionnaire (FOSQ), Epworth Sleepiness Scale (ESS), Pittsburgh Sleep Quality Index (PSQI), and EuroQoL-5-Dimension (EQ-5D) questionnaire between baseline and 12 months after ASV initiation. Additional outcomes were device usage (average h/night) during the follow-up period and changes in respiratory parameters during sleep based on device data (apnea-hypopnea index [AHIflow], apnea index, hypopnea index).

The FOSQ consists of 30 questions (in 5 subsections: activity, vigilance, intimacy, fitness, social life). The potential total score is 5–20, and a total score of ≥ 17.9 is considered normal.^26^ A score change of 1 point is considered to be the minimal clinically important difference (MCID).^27^ The total ESS score is 0–24, with scores of 11–12, 13–15, and 16–24 indicating mild, moderate, and severe excessive daytime sleepiness, respectively. The MCID is estimated to be −2.0 points.^28^

The PSQI contains 19 items that are divided into 7 subcomponent scores, which are combined to provide a global score of 0–21. Higher scores represent more sleep problems (ie, lower sleep quality). A cut-off of 5 score points discriminates poor (> 5) and good sleepers (≤ 5).^29^ A change in score of 3 points is considered to be clinically important.^30^

The EQ-5D includes 5 items that are each rated from 1–5 (1 meaning no problems and 5 meaning severe problems). A 0.08-point change in the index score has been suggested as being clinically relevant.^31^ The EQ-5D visual analog scale indicates the general state of health from 0 (worst possible) to 100 (best possible) (www.euroqol.org). Participants were defined as being symptomatic if they had an FOSQ score < 17.9 and/or an ESS score > 10.

### Follow-up

Demographic and diagnostic data from the polysomnography and respiratory polygraphy were collected during the baseline visit. All questionnaires were completed at baseline and follow-up. The PSQI was discontinued after the run-in phase of the study and was only available for a subset of the total study population. Follow-up visits took place as per clinical routine. At least 1 follow-up visit was documented during the first 12 months of therapy.

### Statistical analyses

There was no formal sample size calculation for the registry. For this subanalysis, the subset of participants who reported baseline use of opioids was included. Continuous variables are presented as the number of observations, mean ± standard deviation, or median (interquartile range) as appropriate. Data were tested for normal distribution using the Kolmogorov-Smirnov test. Categorical variables are summarized using absolute and percentage frequencies. Changes in quality of life parameters are reported as mean or median change from baseline. To test whether changes from baseline were statistically significant, the Wilcoxon matched-pairs test was used. All tests were 2-sided, and a significance level of 5% was used. Statistical analyses were performed using IBM SPSS Statistics 28 (Armonk, New York).

## RESULTS

### Study population

Eighty-six patients with CSA initiated on ASV therapy who reported intake of opioids at baseline were included (ages 65.4 ± 11.9 years, 72% male, body mass index: 31.3 ± 6.4 kg/m^2^). Comorbidities were common, with 87% of participants having ≥ 1 comorbidity; the most common comorbidities were hypertension, atrial fibrillation, coronary artery disease, and diabetes (**Table 1**). All patients had severe sleep apnea (median apnea-hypopnea index: 55 events/h). Baseline FOSQ and ESS scores indicated impaired disease-specific functional status and mild to moderate excessive daytime sleepiness, and 40/49 (82%) of patients who had follow-up questionnaires available were defined as symptomatic at baseline. Baseline sleep quality was poor (median PSQI score of 11), whereas baseline EQ-5D scores indicated problems in performing daily activities and a poor general health status (**Figure S1** in the supplemental material). Forty-six patients were previously treated with continuous or automatically titrating positive airway pressure therapy, and 40 patients had no previous treatment with automatically titrating positive airway or continuous positive airway pressure. Of the 86 eligible participants who had a baseline visit, 57 had a follow-up visit (loss to follow-up was due to death [n = 3], withdrawal of consent [n = 5], and missing follow-up data [n = 21]). The population included in the current analysis was generally comparable to READ-ASV participants who were not using opioids at baseline, except that there was a higher proportion of females and a higher rate of depression in those who take opioids vs those who do not (**Table S1** in the supplemental material).

**Table 1:** Baseline data of adaptive servo-ventilation users with opioid-induced central sleep apnea.

Demographic Characteristics	Participants (n = 86)
Age (years)	65.4 ± 11.9
Male sex, n (%)	62 (72)
Body mass index (kg/m^2^)	31.3 ± 6.4
Symptomatic at baseline, n (%)	40/49 (81.6)
**Comorbidities, n (%)**	
Coronary artery disease	24 (27.9)
Depression	18 (20.9)
Diabetes	22 (25.6)
Heart failure	12 (14.0)
With reduced ejection fraction	0 (0.0)
With midrange ejection fraction	1 (1.2)
With preserved ejection fraction	11 (12.8)
Hypertension	66 (76.7)
Atrial fibrillation	25 (29.1)
Stroke	9 (10.5)
No comorbidity	11 (12.8)
**Diagnostic Sleep Study Findings, (events/h)**	
Apnea-hypopnea index/h	58 ± 27
Central apnea index	17 ± 21
Obstructive apnea index/h	15 ± 19
Mixed apnea index	6 ± 12
Hypopnea index	20 ± 18

Values are mean ± standard deviation or number (%).

### ASV therapy

The residual median apnea-hypopnea index flow during ASV therapy was 1.5 events/h, indicating effective control of SDB. Overall ASV device usage was good (mean 4.8 ± 2.0 h/night), and device usage was ≥ 4 h/night on 66.3% of nights (**Table 2**).

**Table 2:** Device data during adaptive servo-ventilation therapy at 12-month follow-up.

Residual Apnea/Hypopnea Indices (events/h)^a^	Participants (n = 54)
Residual apnea-hypopnea index	2.4 ± 3.9
Residual apnea index	0.6 ± 1.4
Residual hypopnea index	1.8 ± 2.6
**Applied Pressures and Leak**	
Expiratory positive airway pressure (cm H_2_O)	7.0 ± 1.9
Inspiratory positive airway pressure (cm H_2_O)	10.9 ± 2.9
Leak (L/min)	2.53 ± 4.84
Respiration rate (breaths/min)	13.48 ± 3.29
Minute ventilation median (L)	7.04 ± 2.04
**Therapy Usage**	
Daily usage per nights of follow-up (h/night)^b^	4.81 ± 2.02
Daily usage per usage nights (h/night)	6.00 ± 1.59
Proportion of days with usage ≥ 4 hours (%)	66.3 ± 26.3

Values are mean ± standard deviation. ^a^Device data. ^b^Total h/total nights.

### Disease-related quality of life

**Figure 1:**
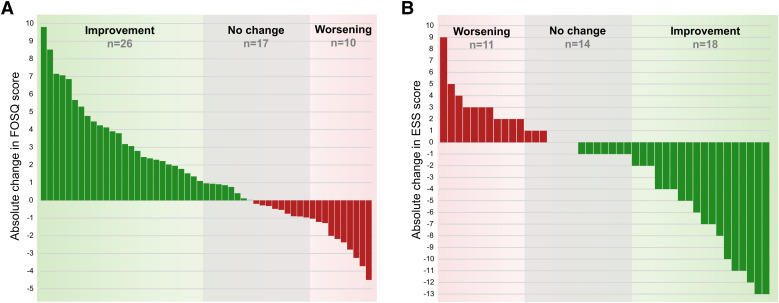
Waterfall plots showing change from baseline to follow-up in Functional Outcomes of Sleep Questionnaire (FOSQ) **(A)** and Epworth Sleepiness Scale (ESS) **(B)** scores.

**Figure 2:**
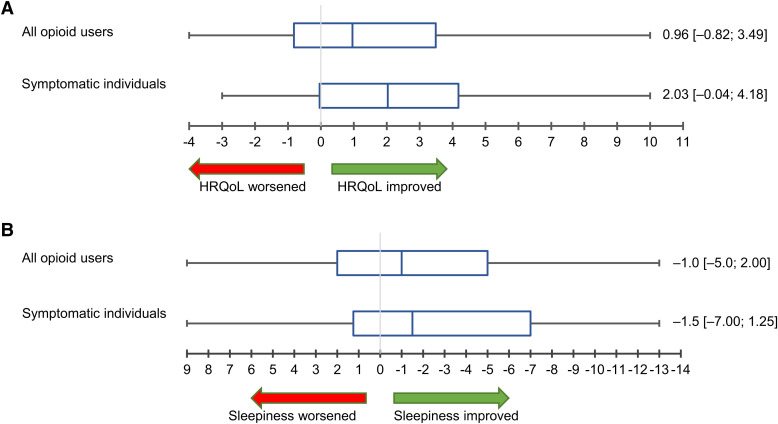
Changes in Functional Outcomes of Sleep Questionnaire (FOSQ) **(A)** and Epworth Sleepiness Scale (ESS) **(B)** scores from baseline to follow-up. Values are median with interquartile range. Data are presented separately for the overall population of people who use opioids and for those using opioids who had symptomatic sleep-disordered breathing at baseline (defined as an FOSQ score < 17.9 and/or an ESS score > 10). HRQoL, health-related quality of life.

FOSQ and ESS scores improved significantly during ASV therapy (**Figure 1**). The median FOSQ score increased by 1.4 points (*P* = .003 vs baseline), and the median ESS score decreased by 3 points (*P* = .029 vs baseline). Changes in both the FOSQ and ESS scores were numerically greater in patients who were symptomatic at baseline (**Figure 2**). The improvement in FOSQ and ESS scores during ASV therapy did not differ significantly between patients previously treated with continuous or automatically titrating positive airway pressure and those who were naive to positive airway pressure therapy (*P* > .05 for all comparisons) (**Table S2** in the supplemental material). There was a slight improvement in sleep quality during ASV therapy (based on the PSQI score), but there were no significant changes from baseline in general quality of life and health status (EQ-5D) (**Figure S1**).

## DISCUSSION

This analysis of data from the European READ-ASV registry found that patients who used opioids treated with ASV therapy for CSA had severe sleep apnea, poor quality of life, and a high burden of sleep-related symptoms. The proportion of symptomatic individuals in the opioid user population (81%) was substantially higher than that in the overall READ-ASV registry population (62%).^22^ Individuals who used opioids were also significantly more likely to be female and have depression compared with those who did not take opioids. Individuals who used opioids with CSA showed good adherence to ASV therapy, and ASV effectively controlled SDB events. In addition, there were significant improvements in disease-specific quality of life and daytime sleepiness during ASV therapy. This reduction in symptom burden was greatest in the subset of patients who were defined as symptomatic at baseline. Non–sleep-related health status was not influenced by ASV therapy.

The changes in health-related quality of life and daytime sleepiness documented during treatment with ASV in individuals who used opioids were clinically relevant as well as statistically significant. The median increase in the FOSQ score was 1.4 points, which is greater than the MCID of 1 point. Similarly, the mean 3-point change in the ESS score was greater than the estimated MCID of 2 points. However, the 2-point reduction in the median PSQI score (indicating an improvement in sleep quality) did not reach the MCID for this measure (3 points).

The improvement in symptoms seen in our study was restricted to disease-specific measures and was not seen for the general quality of life measures. The likely reason for this is that individuals taking opioids are also affected by the overall condition for which they have been prescribed opioids, along with other comorbidities.

Our findings show that eliminating CSA with ASV therapy in people using opioids can improve patient-reported outcome measures in what is a population that experiences significant health impacts of disease. Although individuals taking opioids with CSA may have other causes for sleepiness, we have shown that ASV can contribute to a significant reduction in symptom burden in these individuals, particularly in those with a high symptom burden before treatment initiation. The study participants had multiple health concerns and medications but were highly adherent to ASV, which could be an indirect indication that feeling better while using therapy (ie, self-perceived benefit) motivated them to use the device optimally.

Given the widespread use of opioids in the United States and the considerable number of people taking opioids worldwide, opioid-related central sleep-related breathing disorders will remain an important clinical and economic issue. However, CSA is underdiagnosed and undertreated in this population, and this represents a gap in the cascade of care for opioid use disorders.^32^ There is therefore a need to develop specific care pathways that facilitate the diagnosis and management of CSA of people using opioids. After evaluation with a sleep study and initiation of appropriate therapy (such as ASV), our data suggest that the FOSQ and ESS would be suitable tools to assess the impact of treatment of health-related quality of life during routine follow-up of these patients. However, treatment of SDB represents only one component of the overall health condition of those who take opioids with CSA, as indicated by the lack of change in general quality of life measures in our population. Therefore other approaches and interventions would also be needed to address reduced quality of life in these individuals.

This study has several strengths, including the prospective multinational population that is representative of real-world clinical practice. In addition, it is the largest dataset evaluating the use of ASV in people with opioid-related CSA. However, the population is a subset of a larger registry study.^22^^,^^25^ Some other limitations also need to be considered when interpreting our findings. Although data in the READ-ASV registry were collected prospectively, the study has an observational design making it difficult to account for potential sources of bias. The self-reported nature of the measures used in the study and the act of undergoing follow-up could have contributed to improvements over time, but the key variables assessed were quite specific to sleep-related symptoms. However, the study did lack an objective measure of daytime sleepiness. In addition, we only had information about opioid usage based on the response to a yes/no question at enrollment (data on the type and dosage of opioids used by the patients was not collected in the registry, and this is a significant limitation of our analysis). Another limitation is the lack of sleep study data, which does not allow complete phenotyping of the study population.

In conclusion, ASV was an effective treatment for CSA in people taking opioids and was associated with clinically relevant improvements in disease-specific quality of life and daytime sleepiness. Although requiring confirmation in an appropriately designed clinical trial, the current findings suggest that ASV could be an effective option to counteract the negative effects of opioids on SDB and health-related quality of life.

## DISCLOSURE STATEMENT

All authors have seen and approved the manuscript. Work for this study was performed at the participating sites of the READ-ASV registry in Germany, Switzerland, France, Denmark, Portugal and Spain. This study was funded by ResMed. Editing assistance was provided by Nicola Ryan, independent medical writer, funded by ResMed. The READ-ASV registry was funded by ResMed. J.-L.P. is supported by the French National Research Agency in the framework of the Investissements d’Avenir program (Grant ANR-15-IDEX-02) and the e-Health and Integrated Care and Trajectories Medicine and MIAI Artificial Intelligence (ANR-19- P3IA-0003) chairs of excellence from the Grenoble Alpes University Foundation. He reports lecture fees or conference traveling grants from ResMed, Philips, Jazz Pharmaceuticals, Agiradom, Bastide, and Bioprojet. A.V.B and O.M. are employees of ResMed. H.W. reports lecture/consulting fees from Astra Zeneca, Allergopharma, Bioprojet, Boehringer Ingelheim, Chiesi, GSK, Novartis, Inspire, Jazz Pharmaceuticals, and ResMed, and research support from ResMed and Novartis. R.H. has received speaker or consultancy fees from ResMed, Jazz Pharmaceuticals, Inspire Medical Systems, Sleepres, Bioprojet, Philips, Merck, Nyxoah, Medtronic, Nestlé, and Löwenstein. M.A. has received grant support from ResMed, the ResMed Foundation, and Philips Respironics, and has received lecture and consulting fees from ResMed, Philips Respironics, Inspire Medical Systems, and Zoll outside the submitted work.

## Supplemental Materials

10.5664/jcsm.11652 Supplemental Materials

## ABBREVIATIONS

ASV: adaptive servo-ventilation
CSA: central sleep apnea
EQ-5D: Euro-Qol 5-Dimension
ESS: Epworth Sleepiness Scale
FOSQ: Functional Outcomes of Sleep Questionnaire
MCID: minimal clinically important difference
PSQI: Pittsburgh Sleep Quality Index
READ-ASV: Registry on the Treatment of Central and Complex Sleep-Disordered Breathing with Adaptive Servo-Ventilation
SDB: sleep-disordered breathing

## OPEN ACCESS

Copyright 2025 The Authors. This is an open access article, distributed under the Creative Commons Attribution 4.0 International License. Sharing and adaptation are permitted provided attribution to its original publication in the Journal of Clinical Sleep Medicine is made in accordance with the license.
