# Supplementary material for: Treatment of sleep-disordered breathing in opioid users with adaptive servo-ventilation: a subgroup analysis of the European READ-ASV registry
Source: J Clin Sleep Med. 2025 Jul 1;21(7):1227–32. doi: 10.5664/jcsm.11652 (PMC12225274; doi:10.5664/jcsm.11652)

TABLE S1 Baseline demographic characteristics and comorbidities in users versus non-users of opioids at baseline

	**Opioid users at baseline**		**p-value**
	**Yes (n=86)**	**No (n=715)**	
Age, years	65.4±11.9	67.1±11.9	0.221
Male sex, n (%)	62 (72.1)	627 (87.7)	<0.001
Body mass index, kg/m²	31.3±6.4	30.9±5.3	0.601
Comorbidities, n (%)			
Coronary artery disease	24 (27.9)	209 (29.2)	0.798
Depression	18 (20.9)	74 (10.3)	0.004
Diabetes	22 (25.6)	181 (25.3)	0.957
Hypertension	66 (76.7)	563 (78.7)	0.670
Atrial fibrillation	25 (29.1)	232 (32.4)	0.526
Stroke	9 (10.5)	83 (11.6)	0.753
Heart failure	18 (20.9)	168 (23.5)	0.594

Values are mean ± standard deviation or number of patients (%).

TABLE S2. Changes in Functional Outcomes of Sleep Questionnaire and Epworth Sleepiness Scale scores from baseline to follow-up in patient subgroup based on previously use of continuous or automatically titrating positive airway pressure therapy.

	ESS score		FOSQ score	
	**Baseline**	**Follow-up**	**Baseline**	**Follow-up**
**PAP therapy naïve**				
N	34	21	36	23
Missing	6	19	4	17
Median score (IQR)	9.0 (6.0; 13.8)	8.0 (4.0; 13.5)	15.2 (12.9; 17.6)	17.2 (13.1; 19.3)
**Previous CPAP/APAP**				
N	39	32	44	33
Missing	7	14	2	13
Median score (IQR)	13.0 (7.0; 16.0)	11.0 (5.5; 13.8)	15.3 (11.9; 18.1)	15.7 (13.6; 18.7)
**p-value for between-group comparison**	0.251	0.925	0.420	0.750

APAP, automatically titrating positive airway pressure; CPAP, continuous positive airway pressure; ESS, Epworth Sleepiness Scale; FOSQ, Functional Outcomes of Sleep Questionnaire; IQR, interquartile range.

FIGURE S1 Baseline and follow-up scores for the Pittsburgh Sleep Quality Index (**A**), Euro-Qol 5-dimension (EQ-5D) index (**B**), and the EQ-5D visual analogue scale (**C**). Values are median [interquartile range].

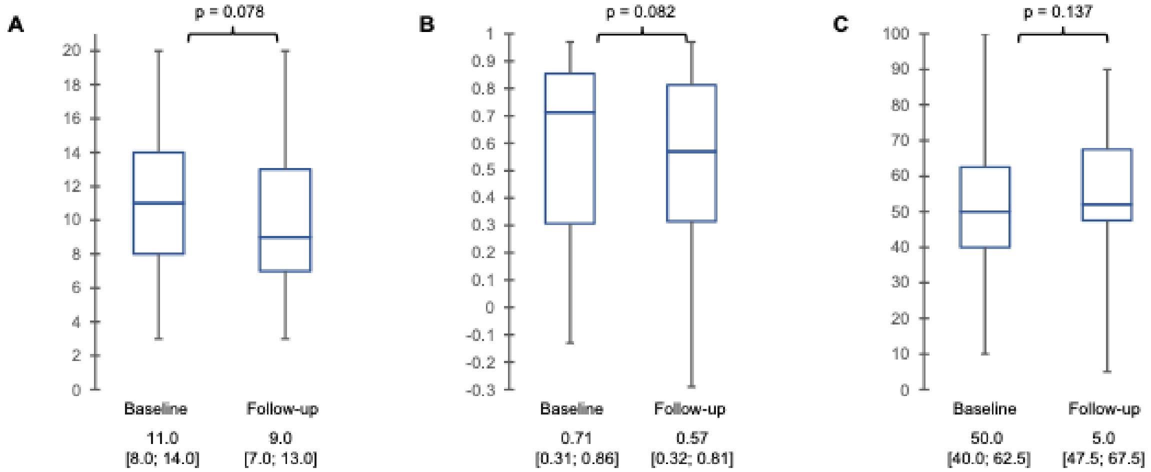

Supplement: Supplemental Materials [file jcsm.11652.sm001.pdf]
